# The complete mitochondrial genome of *Aphaenogaster japonica* (Forel, 1911) (Hymenoptera: Formicidae)

**DOI:** 10.1080/23802359.2022.2095937

**Published:** 2022-07-12

**Authors:** Xin-Yu Luo, Ru-Yi Yin, Xiang-Qin Huang, Yi Luo, Zhao-Min Zhou

**Affiliations:** a Key Laboratory of Southwest China Wildlife Resources Conservation (Ministry of Education), China West Normal University, Nanchong, China; b Key Laboratory of Environmental Science and Biodiversity Conservation (Sichuan Province), China West Normal University, Nanchong, China

**Keywords:** *Aphaenogaster japonica*, Myrmicinae, mitochondrial genome, phylogenetic analysis

## Abstract

*Aphaenogaster japonica* (Forel, 1911) is an omnivorous ant that is widely distributed in eastern Asia. The mitochondrial genome of *A. japonica* reported here was 18,607 bp in length, consisting of 13 protein-coding genes, two ribosomal RNA genes, 22 transfer RNA genes, and a control region. The base composition was AT biased (the GC ratio is 18.9%). With *A. japonica* added, we obtain weak evidence that the sister group of the Stenammini group, including *Aphaenogaster*, is the Myrmicini group. Therefore, the Stenammini and Myrmicini groups may be not a robust monophyletic group, unlike the previous results based on the complete mitochondrial genome.

*Aphaenogaster* is a genus of elongated, slender ants containing 210 extant species distributed all over the world except in sub-Saharan Africa and South America (Kiran et al. 2013; Park et al. 2020). In North America (Giladi 2006; Ness et al. 2009) and East Asia (Higashi et al. 1989), *Aphaenogaster* species are often herb seed dispersers in temperate forests. Based on nuclear gene fragments, *Aphaenogaster* is within the Stenammini group with *Stenamma*, *Messor*, *Goniomma* and *Oxyopomyrmex*, while this group is sister to all myrmicines except the Myrmicini and Pogonomyrmecini groups (Ward et al. 2015). However, based on the complete mitochondrial genome, Stenammini and Myrmicini are grouped (Park et al. 2020; Sang et al. 2022; Yin et al. 2022). *Aphaenogaster japonica* is a common species found in eastern Asia and is currently more abundant in lowlands (Kwon et al. 2014) but is expected to increase in highlands in the future due to global warming (Kwon 2016). To better understand its phylogenetic relationships, we present the complete mitochondrial genome of *A. japonica*.

In this study, *Aphaenogaster japonica* workers were collected from Nanchong City (30°48′54.07″N, 106°4′11.08″E), Sichuan Province, China, in October 2020. All sample collection schemes in this study were approved by the Research Ethics Committee of China West Normal University (approval reference: CWNU2020D002). After morphological identification, we kept the voucher specimens (voucher number: NCAJ202010) in the Key Laboratory of Southwest China Wildlife Resources Conservation of China West Normal University (www.cwnu.edu.cn; Contact: Zhao-Min ZHOU, zhouzm81@gmail.com). We extracted and sequenced the total genomic DNA of *A. japonica* with the Illumina Novaseq sequencing platform (Shanghai Personal Biotechnology Co. Ltd., Shanghai, China). The software A5-miseq v20150522 (Coil et al. 2015) and SPAdes V3.9.0 (Bankevich et al. 2012) were used to carry out the genome de novo assembly. We annotated the mitochondrial genome on the MITOS Web Server (Bernt et al. 2013). Finally, the sequence information of *A. japonica* was submitted to GenBank (accession number: MW915466).

The complete mitogenome of *A. japonica* was 18,607 bp (GC content is 18.9%) in size, consisting of 13 protein-coding genes (PCGs), two rRNA genes, 22 tRNA genes, and a control region. The base composition was 41.0% A, 13.4% C, 5.5% G, and 40.0% T. All PCGs started with ATN (five ATG, four ATT, and four ATA) and ended with TAA. The tRNA size ranged from 52 to 80 bp, which is similar to those of other ant species (54–90 bp) (Idogawa et al. 2021). Four PCGs (ND5, ND4, ND4L, and ND1) and 10 RNAS (tRNA-V, Q, C, Y, F, H, P, L1 and rRNA-L, S) were encoded by the majority strand (J-strand), whereas the others were located on the minority strand (N-strand). The lengths of 12S and 16S rRNA were 936 and 1589 bp, respectively. The gene order of *A. japonica* is consistent with the putative ancestral arrangement of Myrmicinae species (Babbucci et al. 2014; Vieira and Prosdocimi 2019).

Phylogenetic relationships were inferred from 30 ants, including *A. japonica,* and an outgroup species, *Apis mellifera ligustica*. We aligned 13 PCGs and two rRNA genes by MAFFT 7.450 (Katoh and Standley 2013) and concatenated them in PhyloSuite v1.2.2 (Zhang et al. 2020). The best-fit model determined by ModelFinder was GTR + F+I + G4 (Kalyaanamoorthy et al. 2017). The maximum-likelihood and neighbor-joining trees were made by MEGA X (Kumar et al. 2018), and a Bayesian inference tree was constructed using MrBayes 3.2.6 (Ronquist et al. 2012). The phylogenetic trees showed that *A. japonica* and *A. famelica* are grouped with high support values, which may have been caused by high similarity between two mitogenomes in the same genus (Figure 1). Stenammini and Myrmicini were grouped in two of the three trees (Figure 1), which is congruent with the results based on the complete mitochondrial genome (Park et al. 2020; Sang et al. 2022; Yin et al. 2022), but the support values of the monophyletic relationship were much lower.

**Figure 1.**
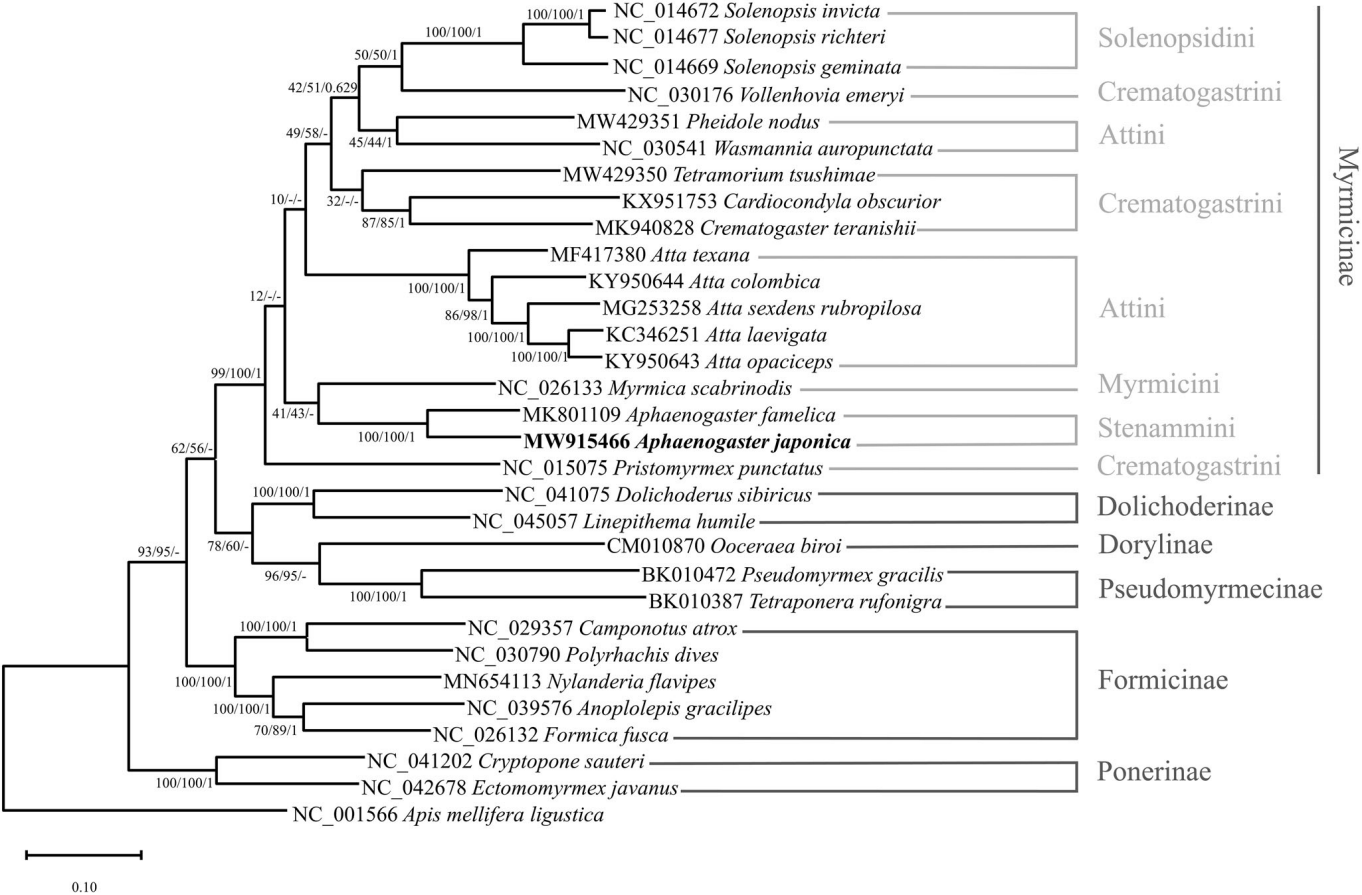
Maximum-likelihood, neighbor-joining, and Bayesian’s inference phylogenetic trees showing phylogenetic relationships among 30 ant species. *Apis mellifera ligustica* was used as an outgroup. A phylogenetic tree was constructed based on the maximum-likelihood phylogenetic tree. The numbers at nodes indicate bootstrap support values for maximum-likelihood and neighbor-joining trees and posterior probability for the Bayesian inference tree, in order.

## Data Availability

The data that support the findings of this study are openly available in GenBank of NCBI at https://www.ncbi.nlm.nih.gov, reference number MW915466. The associated BioProject, SRA, and Bio-Sample numbers are PRJNA814821, SRX14435162, and SAMN26567376, respectively.
